# Developing New Diagnostic Tools Based on SERS Analysis of Filtered Salivary Samples for Oral Cancer Detection

**DOI:** 10.3390/ijms241512125

**Published:** 2023-07-28

**Authors:** Rareș-Mario Borșa, Valentin Toma, Anca Onaciu, Cristian-Silviu Moldovan, Radu Mărginean, Diana Cenariu, Gabriela-Fabiola Știufiuc, Cristian-Mihail Dinu, Simion Bran, Horia-Octavian Opriș, Sergiu Văcăraș, Florin Onișor-Gligor, Dorin Sentea, Mihaela-Felicia Băciuț, Cristina-Adela Iuga, Rareș-Ionuț Știufiuc

**Affiliations:** 1 Dental Medicine Faculty, “Iuliu Hatieganu” University of Medicine and Pharmacy, Pasteur 4, 400349 Cluj-Napoca, Romania; rares.mari.borsa@elearn.umfcluj.ro (R.-M.B.); cristian.dinu@umfcluj.ro (C.-M.D.); dr_brans@yahoo.com (S.B.); horia.opris@umfcluj.ro (H.-O.O.); vacaras_sergiu@yahoo.com (S.V.); florin.onisor@gmail.com (F.O.-G.); mbaciut@umfcluj.ro (M.-F.B.); 2 Research Center for Advanced Medicine—MedFuture, “Iuliu Hatieganu” University of Medicine and Pharmacy, Pasteur 4-6, 400337 Cluj-Napoca, Romania; valentin.toma@umfcluj.ro (V.T.); anca.onaciu@umfcluj.ro (A.O.); moldovan.cristian1994@gmail.com (C.-S.M.); margi.radu@outlook.com (R.M.); diana.cenariu@umfcluj.ro (D.C.); iugac@umfcluj.ro (C.-A.I.); 3 Faculty of Physics, “Babes Bolyai” University, Kogalniceanu 1, 400084 Cluj-Napoca, Romania; gabriela.stiufiuc@ubbcluj.ro; 4 Department of Maxillofacial Surgery and Implantology, “Iuliu Hațieganu” University of Medicine and Pharmacy, Iuliu Hossu 37, 400029 Cluj-Napoca, Romania; 5 County Emergency Hospital Cluj, Clinicilor 3-5, 400006 Cluj-Napoca, Romania; secretariat.chirurgie.cmf@gmail.com; 6 Department of Pharmaceutical Analysis, Faculty of Pharmacy, “Iuliu Hațieganu” University of Medicine and Pharmacy, Pasteur 6, 400349 Cluj-Napoca, Romania; 7 Department of Pharmaceutical Physics-Biophysics, Faculty of Pharmacy, “Iuliu Hatieganu” University of Medicine and Pharmacy, Pasteur 6, 400349 Cluj-Napoca, Romania; 8 TRANSCEND Research Center, Regional Institute of Oncology, 700483 Iasi, Romania

**Keywords:** oral cancer, RAMAN/SERS spectroscopy, biomarkers, plasmonic substrates, multivariate analysis, opiorphin, potassium thiocyanate, uric acid

## Abstract

Cancer still represents one of the biggest challenges in current medical practice. Among different types of cancer, oral cancer has a huge impact on patients due to its great visibility, which is more likely to create social stigma and increased anxiety. New early diagnose methods are still needed to improve treatment efficiency and patients’ life quality. Raman/SERS (Surface Enhanced Raman Spectroscopy) spectroscopy has a unique and powerful potential for detecting specific molecules that can become priceless biomarkers in different pathologies, such as oral cancer. In this study, a batch of saliva samples obtained from a group of 17 patients with oro-maxillofacial pathologies compared with saliva samples from 18 healthy donors using the aforementioned methods were evaluated. At the same time, opiorphin, potassium thiocyanate and uric acid were evaluated as potential specific biomarkers for oro-maxillofacial pathologies using multivariate analysis. A careful examination of SERS spectra collected on saliva samples showed that the spectra are dominated by the vibrational bands of opiorphin, potassium thiocyanate and uric acid. Given the fact that all these small molecules are found in very small amounts, we filtrated all the samples to get rid of large molecules and to improve our analysis. By using solid plasmonic substrates, we were able to gain information about molecular concentration and geometry of interaction. On the other hand, the multivariate analysis of the salivary spectra contributed to developing a new detection method for oral cancer.

## 1. Introduction

Squamous cell carcinoma (SCC) represents over 90% of oral cancers. The incidence of this pathology varies greatly depending on the geographical area, ranking among the top 10 most common types of cancer worldwide [1]. Patients diagnosed with SCC of the oral cavity are generally older men from disadvantaged socioeconomic backgrounds. The lack of prominent symptoms, characterized by tolerable and mild pain, leads to delayed presentation of patients to specialized services, resulting in a reserved prognosis.

Currently, the development of strategies aimed at addressing these shortcomings is urgently necessary. Prevention through accurate information and screening can represent two starting points in achieving early diagnosis methods.

Saliva is a true pioneer in the field of liquid biopsy due to its complexity compared with other biological fluids (urine, blood and their derivatives). Furthermore, saliva reflects the level of biomarkers in real time, similar to plasma composition, for example [2]. The major advantages of using this fluid are numerous and noteworthy for both researchers and current medical professionals: noninvasive and painless collection, reduced costs and easy storage. Additionally, it should be noted that salivary samples can identify the “parent molecules” of certain substances, unlike urine, which is abundant in their metabolites [3]. These characteristics provide the possibility of conducting large-scale studies with maximum benefits at minimal costs. Thus, saliva can be used in the future to assess and monitor the overall status of the body.

This biofluid mainly contains the secretions of major paired salivary glands (parotid, submandibular and sublingual) and minor glands of the oral cavity [4]. The formation of saliva is also influenced to a lesser extent by crevicular gingival fluid and oral mucosa exudate [5].

Saliva is a colorless, odorless liquid with a relative density of 1.004–1.009 g/cm^3^ and a pH ranging from 6.6–7.1 [5]. Under normal conditions, approximately 600 mL of saliva is secreted daily [5]. Salivary flow can be influenced by factors such as body posture or ambient light intensity, with a reduction of up to 40% in a dark environment [3].

Its composition is complex and varies depending on the collection method, time of day, circadian rhythm of the subject and season [3]. In addition to its overwhelming water content (99%) [5], saliva also contains organic and inorganic dry residue, including electrolytes, proteins and epithelial cells from the oral mucosa [4]. The organic matter of saliva includes salivary amylase, mucopolysaccharides, mucin, lysozyme, urea, ammonium, uric acid, glucose, cholesterol, fatty acids, neutral lipids, glycolipids, amino acids, steroid hormones, lecithin, glycoproteins, peroxidase, lactoferrin [5] and opiorphin [6]. The inorganic matter consists of elevated concentrations of various electrolytes (${\mathrm{Na}}^{+}$, ${\mathrm{Cl}}^{-}$, ${\mathrm{Ca}}^{2+}$, $K^{+}$, ${\mathrm{HCO}}_{3}^{-}$, $H_{2}{\mathrm{PO}}_{4}^{-}$, $F^{-}$, $I^{-}$, ${\mathrm{Mg}}^{2+}$) and the thiocyanate ion (${\mathrm{SCN}}^{-}$) [5].

Opiorphin is an endogenous compound first isolated from human saliva. Etymologically, the substance’s name derives from combining the words opium and morphine, which hints at its properties [6]. Chemically, opiorphin ($C_{29}H_{48}N_{12}O_{8}$) is a tetrapeptide composed of four amino acids: glutamine (Gln), arginine (Arg), phenylalanine (Phe) and serine (Ser), with the following composition: C (50.28%), H (6.98%), N (24.26%) and O (18.48%). The molecule of this substance exhibits high polarity and great water solubility, making it inherently soluble in saliva [6]. A higher concentration of opiorphin has been found in salivary and lacrimal secretions compared with other biological fluids, suggesting a possible involvement of this substance in oro-facial pain perception [7]. The endogenous nature of opiorphin contributes to the body’s better tolerance to opioids, partially reducing or even completely preventing the side effects of this class of medications such as dependence or constipation. Thus, there are possibilities for the future use of this compound in pain management strategies [6].

Potassium thiocyanate (KSCN) is an inorganic compound with a defensive role. It is physiologically secreted in the oral cavity in saliva and serves as a biomolecule with antioxidant properties in the immune system. Similar to opiorphin, the highest concentration of thiocyanate among all biological fluids is found in saliva [8]. Salivary thiocyanate ions undergo an oxidation process in the presence of hydrogen peroxide, catalyzed by the enzyme lactoperoxidase, thereby playing an antibacterial role. This could serve as an alternative carioprophylactic measure to fluoridation [9]. It is also worth mentioning that a more pronounced secretion of potassium thiocyanate has been observed in smokers compared with nonsmokers, due to a defensive antioxidation mechanism [10]. Opiorphin and potassium thiocyanate are metabolites with significant diagnostic potential.

The level of these compounds can be investigated using chromatography techniques combined with mass spectrometry, particularly in the case of KSCN. A major disadvantage of these techniques is the interference caused by the presence of other anions. An alternative approach is the use of Raman/SERS spectroscopy due to its high sensitivity and specificity [3,11].

Uric acid is the final degradation product of the purine’s metabolism. A chain of chemical reactions take place: purine is metabolized to hypoxanthine, then xanthine de-hydrogenase oxidizes to xanthine and then xanthine to uric acid. Serum uric acid in most mammals is extremely low due to the presence of uricase, which oxidizes uric acid to allantoin. Primates, such as humans, that have lost uricase have several mechanisms to regulate serum uric acid levels. One is the increased excretion of uric acid by uric acid transporters, and the other is the increased activity of hypoxanthine-guanine phosphoribosyl transferase, which recycles purines [2].

Uric acid plays an important antioxidant role in extracellular space. Humans do not have the enzyme required for ascorbic acid synthesis, which also possesses strong antioxidant properties. Thus, uric acid compensates for the aforementioned role of ascorbic acid [12]. In addition to this, uric acid plays an oxidative role in the intracellular space due to xanthine oxidase activity, increase in NADPH oxidase and mitochondrial reactive oxygen species (ROS) production (a consequence of mitochondrial injury) [2].

On the other hand, blood samples and tissue biopsy collection have a series of disadvantages, such as pain, fear, invasiveness and bleeding. Nowadays, more precise and efficient diagnosis methods are required in order to balance the inconveniences of the aforementioned ones.

Raman spectroscopy gained its notoriety in science fields over time. This technique is based on inelastic scattering of photons from monochromatic laser radiation. Therefore, molecules from small amounts of samples can be identified (molecular fingerprint). One major inconvenience of this method is represented by the incapacity of performing analysis with a low concentration of molecules.

Surface-enhanced Raman spectroscopy (SERS) was developed in order to wipe out the disadvantages of Raman spectroscopy. It utilizes substrates with enhanced plasmonic properties that allow for the amplification of Raman vibrational modes. In this regard, solid plasmonic substrates based on colloidal silver or gold nanoparticles could represent a new class of nanoobjects with broad applicability in biomedicine for the development of diagnostic strategies [3,11,13,14]. It is worth mentioning SERS analysis can also provide accuracy and sensibility in the detection of some molecular changes at plant cell level [15,16,17,18]. This tremendous capacity to enhance the Raman signal of the analytes holds a great promise for a huge number of biomedical applications.

In this paper, we report the SERS analyses of salivary samples collected from 17 oral cancer patients and 18 controls. Given the fact that the biomarkers present low molecular weight, all the samples included in this study were filtered using 3 kDa membrane tubes. The major vibrational bands observed in the spectra were assigned to opiorphin, intermediate species of uric acid metabolism (e.g., purine, hypoxanthine, xanthine and allantoin) and potassium thiocyanate. By performing a comprehensive multivariate analysis (PCA-LDA) of the SERS spectra collected on filtered saliva, we were able to discriminate the patient group from the controls with high accuracy, sensitivity and specificity.

## 2. Results

### 2.1. Subject Data and Classification

Twenty-one oral cancer patients and twenty healthy donors were initially enrolled in the study. One sample of saliva was collected from each donor. After samples processing, we consider excluding four samples from the patient group and two samples from the control group due to sample irregularities of insufficient volume and low quality. Therefore, in this study, seventeen oral cancer patients and eighteen healthy donors were included. The clinical data of the patients’ cohort are presented in Table S1. The healthy donors’ group data are presented in Table S2.

### 2.2. Raman/SERS Analysis of Saliva Samples

**Figure 1:**
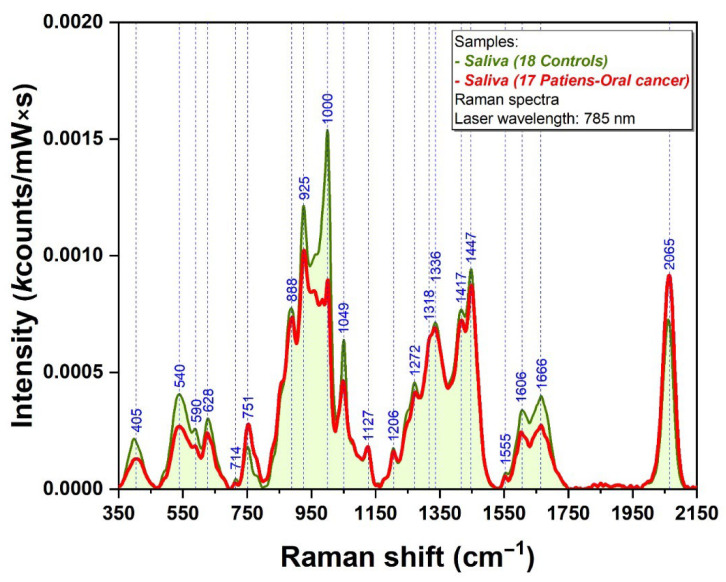
Mean Raman spectra of filtered salivary samples collected from controls (green spectrum) and oral cancer patients (red spectrum), using an excitation wavelength of 785 nm. The main vibrational peaks are marked for clarity.

It was previously shown in the literature that the Raman spectra of biofluids are dominated by the vibrational bands of the proteins [19]. This is the reason why all salivary samples included in this study were filtered using 3 kDa filters, capable of removing the vast majority of proteins present in the samples. In Figure 1, we plotted the mean Raman spectra collected from controls (green spectrum) and from cancer samples (red spectrum), respectively. The individual Raman spectra are plotted in Figure S1.

Differences between the intensities of some vibrational bands can be observed by careful analysis of control and cancer spectra. The biggest difference can be detected in the case of a 1000 cm^−1^ peak. The following peaks belonging to controls have a bigger intensity than those belonging to oral cancer patients: 405, 540, 590, 628, 714, 888, 925, 1000, 1049, 1206, 1272, 1318, 1417, 1447, 1555, 1606 and 1666 cm^−1^. For the other peaks, the situation is the opposite (751 and 2065 cm^−1^).

**Figure 2:**
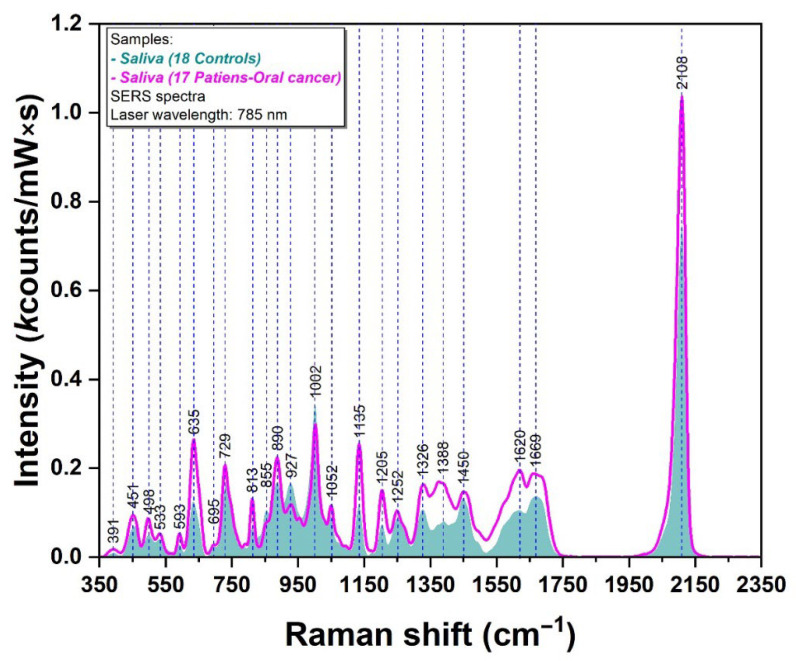
Mean SERS spectra of filtered salivary samples collected from controls (jade spectrum) and cancer patients (magenta spectrum), using an excitation wavelength of 785 nm. The main vibrational peaks are marked for clarity.

The SERS spectra of the same samples are presented in Figure 2. Over time, our group has developed different strategies for the production of plasmonic substrates that can be used for SERS analysis of biomolecules. The major advantage of using solid plasmonic substrates for SERS analysis of biofluids is represented by the high degree of experimental reproducibility generated by these substrates. In a previous paper, we explained in detail the synthesis steps of such substrates that have been further employed for the analysis of blood plasma samples collected from breast cancer patients [20]. The spectra presented here were recorded using a solid plasmonic substrate based on silver nanoparticles developed in our group, capable of considerably improving experimental reproducibility [20]. The characterization of the silver nanoparticles included transmission electron microscopy, atomic force microscopy and calculation of enhancement factor. These results are presented in the Supplementary Materials File, along with Figures S2–S4.The individual SERS spectra collected on the filtered salivary samples are shown in Figure S2.

As it can be seen in the figure, the spectra are dominated by the 2108 cm^−1^ peak. Only three peaks belonging to controls have a bigger intensity than those belonging to oral cancer patients: 855, 927 and 1002 cm^−1^. For the other peaks, the situation is the opposite (391, 451, 498, 533, 593, 635, 695, 729, 813, 890, 1052, 1135, 1205, 1252, 1326, 1388, 1450, 1620, 1669 and 2108 cm^−1^).

### 2.3. Multivariate Analysis of the SERS Spectra

**Figure 3:**
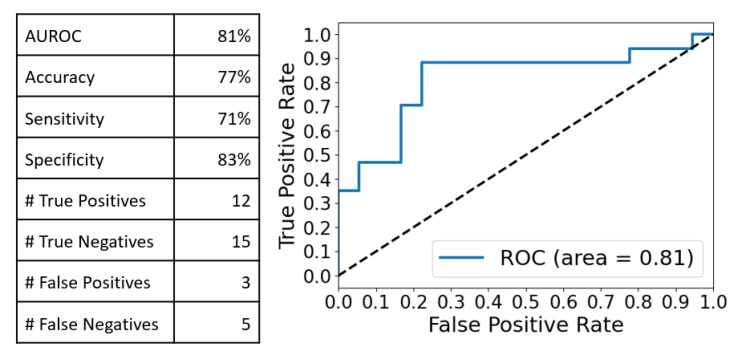
Classification performance obtained after the PCA-LDA analysis of the full range SERS spectra using 5 PC components. AUROC—the area under the receiver operating characteristic. 77% accuracy, 71% sensitivity and 83% specificity were obtained.

**Figure 4:**
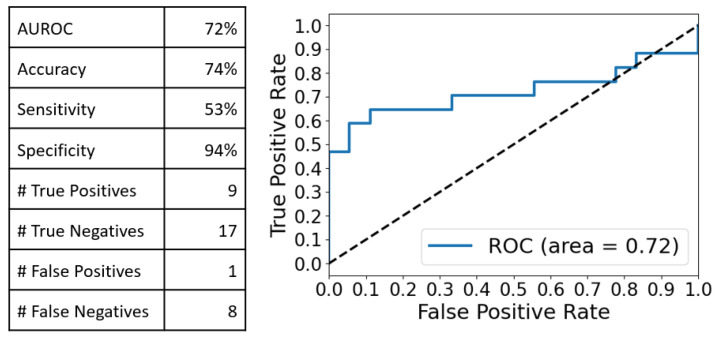
Classification performance obtained after the PCA-LDA analysis of the SERS spectra in the 550–1250 cm^−1^ spectral interval using 5 PC components: 74% accuracy, 53% sensitivity and 94% specificity were obtained.

**Figure 5:**
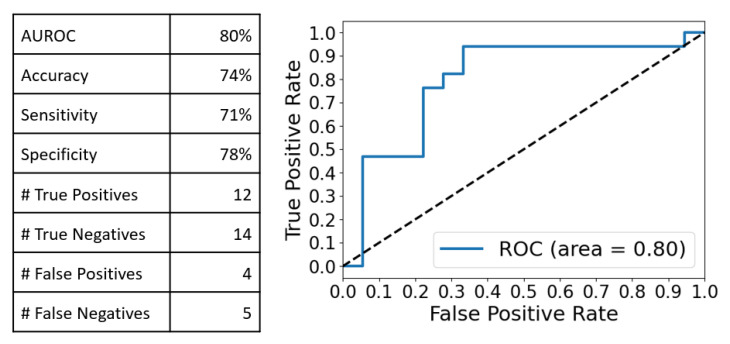
Classification performance obtained after the PCA-LDA analysis of the SERS spectra in the 1950–2150 cm^−1^ spectral interval using 5 PC components: 74% accuracy, 71% sensitivity and 78% specificity were obtained.

**Figure 6:**
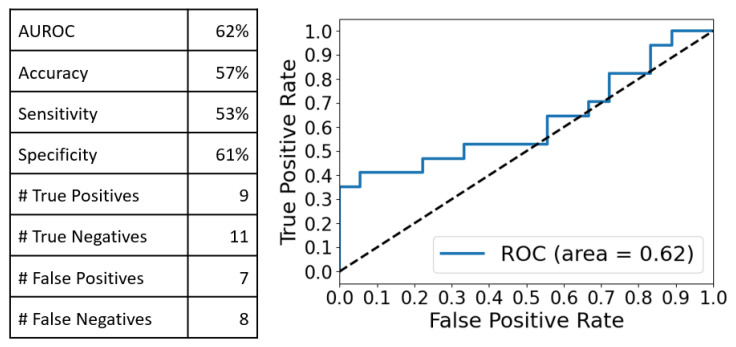
Classification performance obtained after a PCA-LDA analysis of the full range Raman spectra using 5 PC components: 57% accuracy, 53% sensitivity and 61% specificity were obtained.

In order to assess the effectiveness of a multivariate approach in distinguishing be-tween the two groups (patients, n = 17 vs. control, n = 18), we conducted a combined analysis, known as Principal Component Analysis–Linear Discriminant Analysis (PCA-LDA), on all SERS spectra. PCA was employed to transform the data into a lower-dimensional space, maximizing the explained variance in the new dimensionality. At 16 principal components, PCA achieved 99% explained variance. However, PCA-LDA yielded an overfit model (97% training accuracy vs. 83% Leave-One-Out Cross-Validation (LOOCV) accuracy). Therefore, we used five PCA components, which account for 90% of the total variance and are less overfit in the PCA-LDA analysis (85% training accuracy vs. 77% LOOCV accuracy). The results are presented in the following classification performance (Figure 3):

Since analyzing smaller segments of the spectra reduces the dimensionality even more and allows for more focused and interpretable results, as well as isolating more informative regions with higher signal-to-noise ratios, we went further by running a PCA-LDA on the intervals 550–1250 cm^−1^ and 1950–2150 cm^−1^. Using five PCA components, this resulted in 90% and 99.75% explained variance, respectively, and the following classification performances (Figure 4 and Figure 5):

The same analysis was performed for the Raman spectra. PCA achieved 99% explained variance with 17 principal components, where the first 3 components explained 71.9% of the total variance. Using PCA-LDA with five components (84% explained variance, 74% training accuracy vs. 57% LOOCV accuracy), we obtained the following classification performance (Figure 6):

## 3. Discussion

SERS is a surface technique that strongly enhances the vibrational signature (molecular fingerprint) of the molecules which are located in the vicinity of the plasmonic substrate (less than 10 nm). Having in mind that proteins, which are abundant in all biological fluids, have the tendency to “occupy” all these regions, we decided, prior to any experimental measurements, to filtrate all the salivary samples included in this study.

The spectra represented in Figure 1 highlight this protein removal process, since the bands associated with different vibrational modes specific to proteins (located around 1350/Amide III and 1650 cm^−1^/Amide I) do not dominate the Raman spectra anymore, as was the case for unfiltered salivary samples [8]. Moreover, the main difference observed between control and cancer samples was located at ~1000 cm^−1^. In the literature, this peak was constantly assigned to the symmetric ring breathing mode of phenylalanine (Phe) [21].

The SERS analysis (Figure 2), performed on solid plasmonic substrates, shows that small molecules like thiocyanate (SCN) dominate the spectra. We previously assigned the most intense vibrational peak of both SERS spectra included in Figure 2 (2108 cm^−1^) to a specific vibrational band of SCN. In a previous study, we demonstrated that the intensity of this peak can be considerably increased after X-ray irradiation, suggesting that SCN can be considered as a possible biomarker for different diseases [8].

Besides the 2108 cm^−1^ band, the SERS spectra of filtered salivary samples are dominated by other peaks: 635, 729, 813, 890 1002 and 1135 cm^−1^. The second most intense peak is 1002 cm^−1^ (assigned to Phe).

One has to note here that the intensity of the vast majority of the vibrational bands is higher in the case of cancer samples with respect to controls. Only three bands (855, 927 and 1002 cm^−1^) have a different behavior. For a better assessment of these vibrational bands, the SERS spectra of opiorphin, uric acid and hypoxanthine were also recorded (Figure S5). Opiorphin (OPI—an opioid peptide present in saliva) is a compound that has a major role in oro-facial pain perception. From a chemical point of view, this structure is dominated by the presence of Phe. On the other hand, hypoxanthine (HX) and uric acid (UA) are two molecules present in biofluids that have been identified in several SERS studies involving the use of blood plasma and serum. They have a strong affinity for the plasmonic substrates employed in these studies. The assignment of these major vibrational bands recorded on salivary samples are presented in Table S3.

As it can be seen in the figure, the SERS spectra of filtered salivary samples are dominated by the peaks that can be assigned to these four molecules: thiocyanate, OPI, UA and HX. The second most intense peak (1002 cm^−1^), has been assigned so far to a ring breathing mode of phenylalanine, but phenylalanine is a constituent of OPI. As such, we believe that it can be assigned to OPI, a molecule which is very present in human saliva.

By comparing the SERS spectra of cancer patients and controls, one can observe that the only peaks that show a reduced intensity in the case of cancer patients can be assigned to OPI. All the other peaks that present an increased intensity for cancer patients can be assigned to HX and UA. These observations could indicate that in the case of oral cancer, the concentration of salivary OPI decreases, causing an increase in pain perception. Meanwhile, the concentrations of UA and HX could increase in oral cancer samples.

These observations were confirmed by the multivariate analysis (MVA) performed on the integral Raman and SERS spectra. The PCA-LDA analysis yielded very good discrimination results in the case of SERS spectra as compared with the Raman ones (Accuracy:Sensitivity:Specificity of 77%:71%:83% vs. 57%:53%:61%). Moreover, by comparing these values with those obtained when the MVA was performed on two distinct spectral intervals (550–1250 cm^−1^ and 1950–2150 cm^−1^), one can conclude that the best discrimination results between cancer and controls are obtained in the case of a PCA-LDA analysis performed on the full-range SERS spectra, using only five PC components.

## 4. Materials and Methods

### 4.1. Sample Collection

Between August 2022 and April 2023, a total of 17 samples were collected from the group of subjects with oral cancer, along with 18 samples from the control group. The collection was carried out at the Clinic of Oral and Maxillofacial Surgery of the Emergency County Hospital in Cluj, in accordance with the regulations and after obtaining approvals from the Ethics Committees of the aforementioned institution (229/27 February 2022). Additionally, the study was approved by the Ethics Committee for Scientific Research of the “Iuliu Hațieganu” University of Medicine and Pharmacy in Cluj-Napoca (AVZ227/25 July 2022).

Inclusion criteria:

- Adult patients with malignant pathologies in the oro-maxillofacial area.
- Healthy adult subjects (for the control group).

Exclusion criteria:

- Minor patients or adults without pathologies in the oro-maxillofacial area.
- Subjects who are not healthy (not eligible for the control group).

The collection itself involved obtaining 2 mL of saliva per sample using 100 mL graduated urine culture containers (Nerbe Plus, Winsen, Germany). These containers were chosen due to their wide opening, which increased the accuracy of collection and the comfort of the patients during the donation process. It should be noted that subjects were prohibited from eating, drinking, brushing their teeth, chewing gum, smoking, undergoing biopsy for histopathological analysis or smoking in the vicinity or during saliva collection.

The collected samples were aliquoted in 1.5 mL low-bind tubes (Eppendorf, Hamburg, Germany), which were then stored at −80 °C.

### 4.2. Sample Processing

The first step of the protocol included several ultrapure water (ELGA Labwater from PURELAB Chorus, Buckinghamshire, UK) washes of the 3 kDa cellulose membrane filter tubes (Amicon Ultra-0.5 Centrifugal Filter Unit, Sigma Aldrich, Hamburg, Germany) in order to obtain a glycerin-free media. This step was performed by centrifugation at 14,000× *g* for 15 min.

The saliva samples were let to thaw at 4 °C, and then a centrifugation step at 14,000× *g* for 1 min was carried out in order to precipitate the salivary debris. Then, the supernatant was filtered using 3 kDa filter tubes at 14,000× *g* for 10 min at a temperature of 4 °C.

A total of 35 saliva samples were processed according to the aforementioned proto-col as follows: 17 samples from the patient group and 18 samples from the control group.

### 4.3. SERS Substrate Preparation

SERS substrate consisted of plasmonic silver nanoparticles synthesized according to a method developed by Leopold and Lendl [22], which was widely explored by our group in various studies [20,23,24,25,26,27]. The synthesis procedure refers to the reduction in silver nitrate ions to silver nanoparticles in the presence of hydroxylamine molecules. Briefly, in 80 mL ultrapure water (18.2 MΩ × cm, ELGA Labwater from PURELAB Chorus, Buckinghamshire, UK), 5 mL of NH_2_OH·HCl 30 mM and 5 mL of NaOH 63.5 mM were added under 400 rpm stirring conditions at room temperature. Then, 10 mL of AgNO_3_ 10 mM was incorporated under the same stirring conditions for 5 min until a brown to yellowish coloration occurred. The silver colloid was supposed to tangential flow filtration (TFF, Pall Corporation, New York, NY, USA) using a 5 kDa TFF microcapsule for purification and concentration purposes. Moreover, a rigorous physical characterization of the colloid was performed prior to SERS measurements. These filtered nanoparticles were mixed with the saliva samples, deposited on a CaF_2_ glass and let dry in order to produce a robust plasmonic substrate capable to generate reproducible SERS spectra.

### 4.4. Raman/SERS Measurements

The sample preparation for SERS measurements was based on an incubation step by mixing 1 µL of filtered saliva and 1 µL of concentrated plasmonic silver nanoparticles [20]. This mixture was carefully poured on a fresh CaF_2_ Raman-grade glass (Crystran, Poole, UK) and allowed to dry at room temperature. After 30 min, the samples were ready to be measured. All the samples were prepared using the same batch of silver colloids, and inter- and intrabatch reproducibility was assessed for several other biological fluids [8,20,24,27].

The Raman and SERS spectra were obtained using an inVia Reflex Raman confocal multilaser spectrometer (Renishaw™, Wotton-under-Edge, UK) with a spectral resolution of 2 cm^−1^. To calibrate the wavelength, an internal silicon reference was used. All the spectra presented in this paper were captured using a 50× objective with a numerical aperture (N.A) of 0.85. For excitation, a 785 nm diode laser from Renishaw (UK) was employed. The laser power, measured at the sample surface, was approximately 65 mW for Raman measurements and 2 mW for SERS measurements. The acquisition time was set to 20 s. The spectrograph featured a 600 lines/mm grating and a charge-coupled device (CCD) camera. Baseline correction was applied to all spectra to eliminate the fluorescence background. Each spectrum represented an average of 30 spectral acquisitions from different positions across the entire dried sample area.

### 4.5. Data Analysis

Data collection and spectral preprocessing, such as cosmic ray removal and baseline correction, were performed using WiRE 4.2 software from Renishaw plc (Gloucestershire, UK). Raman and SERS spectra were processed using Origin Pro 2019 software. For each sample, the final spectrum represents the average number of spectra after fluorescence background and cosmic ray removal.

In order to assess if there is a separation between these two groups of samples (oral cancer vs. healthy donors), we performed a Principal Component Analysis combined with Linear Discriminant Analysis (PCA-LDA) on all the SERS spectra.

## 5. Conclusions

In this study, we report a Raman/SERS analysis of filtered salivary samples collected from oral cancer patients and controls. This analysis allowed a new assignment of the most intense vibrational bands recorded in the SERS spectra of salivary samples. The presence of opiorphin in salivary samples was experimentally proven for the first time. It is also suggested that opiorphin could be considered as a potent biomarker for oral cancer diagnosis. On the other hand, the MVA analysis performed on Raman and SERS spectra recorded from filtered salivary samples suggested that it could be used for developing a new oral cancer detection method with very good accuracy (57% and 77%), sensitivity (53% and 71%) and specificity (61% and 83%).

## Data Availability

All detailes regarding patients’ database and many more significant information can be found in Supplementary Materials Files. If more information is needed, please contact the coresponding author.

## Supplementary Materials

The following supporting information can be downloaded at https://www.mdpi.com/article/10.3390/ijms241512125/s1. References [28,29,30,31,32,33,34,35,36,37,38,39,40,41,42,43,44,45,46,47,48,49] are cited in the supplementary materials.
