# Supplementary material for: Developing New Diagnostic Tools Based on SERS Analysis of Filtered Salivary Samples for Oral Cancer Detection
Source: Int J Mol Sci. 2023 Jul 28;24(15):12125. doi: 10.3390/ijms241512125 (PMC10418512; doi:10.3390/ijms241512125)
Supplement: Supplementary file 1 [file ijms-24-12125-s001.zip › ijms-2515217-supplementary.pdf]

# Developing new diagnostic tools based on SERS analysis of filtered salivary samples for oral cancer detection

**Rareș-Mario Borșa [1,2], Valentin Toma [2], Anca Onaciu [2], Cristian-Silviu Moldovan [2], Radu Mărginean [2], Diana Cenariu [2], Gabriela-Fabiola Știufiuc [3], Cristian-Mihail Dinu [1,4,5], Simion Bran [1,4,5], Horia-Octavian Opriș [1,4,5], Sergiu Văcăraș [1,4,5], Florin Onișor-Gligor [1,4,5], Dorin Sentea [5], Mihaela-Felicia Băciuț [1,4,5], Cristina-Adela Iuga [2,6] and Rareș-Ionuț Știufiuc [2,7,8*]**

[1] Dental Medicine Faculty, "Iuliu Hatieganu" University of Medicine and Pharmacy, Pasteur 4, 400349, Cluj-Napoca, Romania; RARES.MARI.BORSA@elearn.umfcluj.ro;

[2] Research Center for Advanced Medicine - MedFuture, "Iuliu Hatieganu" University of Medicine and Pharmacy, Pasteur 4-6, 400337 Cluj-Napoca, Romania; valentin.toma@umfcluj.ro, anca.onaciu@umfcluj.ro; moldovan.cristian1994@gmail.com; margi.radu@outlook.com; diana.cenariu@umfcluj.ro; iugac@umfcluj.ro; rares.stiufiuc@umfcluj.ro;

[3] Faculty of Physics, "Babes Bolyai" University, Kogalniceanu 1, 400084 Cluj-Napoca, Romania; gabriela.stiufiuc@ubbcluj.rop;

[4] Department of Maxillofacial Surgery and Implantology, "Iuliu Hațieganu" University of Medicine and Pharmacy, Iuliu Hossu 37, 400029 Cluj-Napoca, Romania; mbaciut@yahoo.com; cristian.dinu@umfcluj.ro; dr_brans@yahoo.com; horia.opris@umfcluj.ro; vacaras_sergiu@yahoo.com; florin.onisor@gmail.com;

[5] County Emergency Hospital Cluj, Clinicilor 3-5, 400006 Cluj-Napoca, Romania; secretariat.chirurgie.cmf@gmail.com

[6] Department of Pharmaceutical Analysis, Faculty of Pharmacy, "Iuliu Hațieganu" University of Medi-cine and Pharmacy, Pasteur 6, 400349 Cluj-Napoca, Romania;

[7] Department of Pharmaceutical Physics-Biophysics, Faculty of Pharmacy, "Iuliu Hatieganu" University of Medicine and Pharmacy, Pasteur 6, 400349 Cluj-Napoca, Romania;

[8] TRANSCEND Research Center, Regional Institute of Oncology, 700483 Iasi, Romania

* Correspondence: rares.stiufiuc@umfcluj.ro; Tel.: +40726340278

**Supplementary Table S1.** Demographic data and cancer-related information of the oral cancer patients enrolled in the study.

| Number | Age (years) | Sex | Urban (U)/rural (R) | Smoker | Special Diet | General pathologies |
|---|---|---|---|---|---|---|
| 1 | 66 | F | U | NO | NO | YES |
| 2 | 58 | M | U | YES | NO | YES |

| | | | | | | |
|---|---|---|---|---|---|---|
| 3 | 48 | M | U | NO | NO | YES |
| 4 | 71 | F | U | NO | NO | YES |
| 5 | 66 | F | R | YES | YES | YES |
| 6 | 49 | M | R | YES | NO | YES |
| 7 | 75 | F | R | NO | NO | YES |
| 8 | 52 | M | U | YES | NO | YES |
| 9 | 64 | M | R | YES | NO | YES |
| 10 | 60 | M | U | YES | NO | YES |
| 11 | 61 | M | M | YES | NO | YES |
| 12 | 73 | M | U | YES | NO | YES |
| 13 | 65 | M | R | YES | NO | YES |
| 14 | 66 | M | R | YES | NO | YES |
| 15 | 45 | F | R | NO | NO | NO |
| 16 | 75 | F | U | YES | YES | YES |
| 17 | 71 | M | U | YES | NO | YES |

**Supplementary Table S2.** Demographic data of controls enrolled in the study.

| Number | Age (years) | Sex | Urban (U)/rural (R) | Smoker | Diet | General pathologies | Medication |
|---|---|---|---|---|---|---|---|
| 1 | 31 | F | R | NO | NO | NO | NO |
| 2 | 33 | F | R | NO | NO | NO | NO |
| 3 | 33 | M | R | NO | NO | NO | NO |
| 4 | 28 | M | U | YES | NO | NO | NO |
| 5 | 22 | M | U | NO | NO | NO | NO |
| 6 | 24 | M | U | NO | NO | NO | NO |
| 7 | 21 | M | U | YES | NO | NO | NO |
| 8 | 24 | F | U | NO | NO | NO | NO |
| 9 | 24 | F | U | NO | NO | NO | NO |
| 10 | 24 | M | U | NO | NO | NO | NO |
| 11 | 24 | M | U | NO | NO | NO | NO |
| 12 | 25 | M | U | NO | NO | NO | NO |
| 13 | 24 | F | U | NO | NO | NO | NO |
| 14 | 26 | M | U | NO | NO | NO | NO |
| 15 | 26 | F | U | YES | NO | NO | NO |
| 16 | 24 | F | U | YES | NO | NO | NO |
| 17 | 24 | F | U | NO | NO | NO | NO |
| 18 | 25 | F | U | NO | NO | NO | NO |

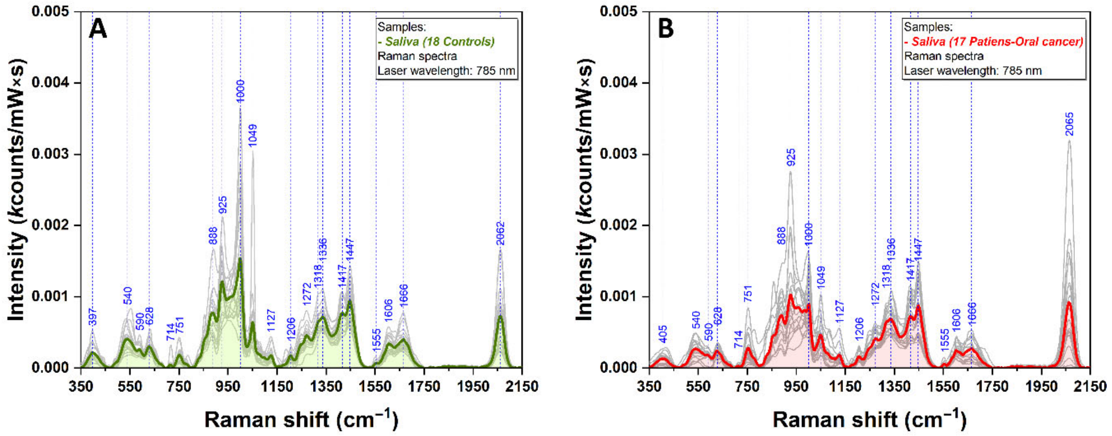


**Figure S1.** Mean Raman spectra of filtered salivary samples collected from control (A, green) and oral cancer (B, red) group samples using a 785 nm excitation laser.

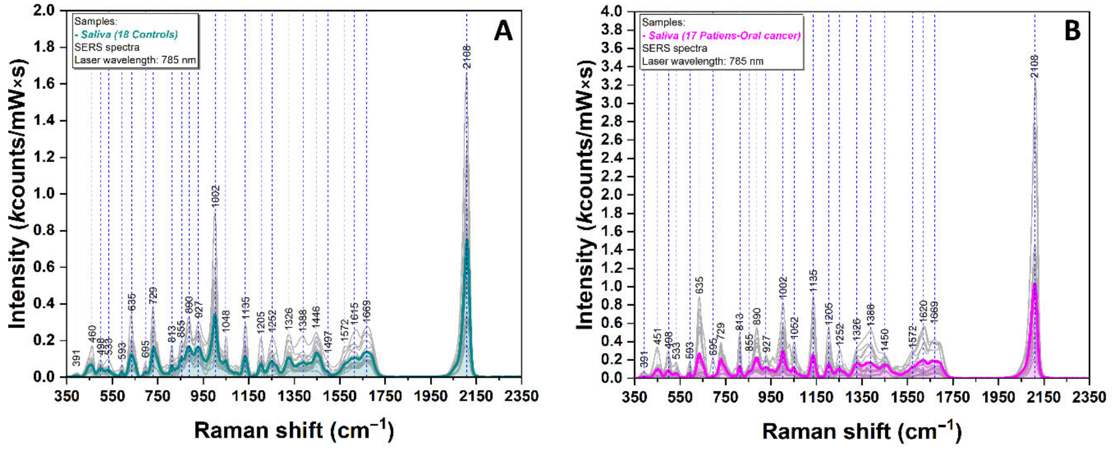


**Figure S2.** Mean SERS spectra of filtered salivary samples collected from control (A, jade) and oral cancer (B, magenta) group samples using a 785 nm excitation laser.

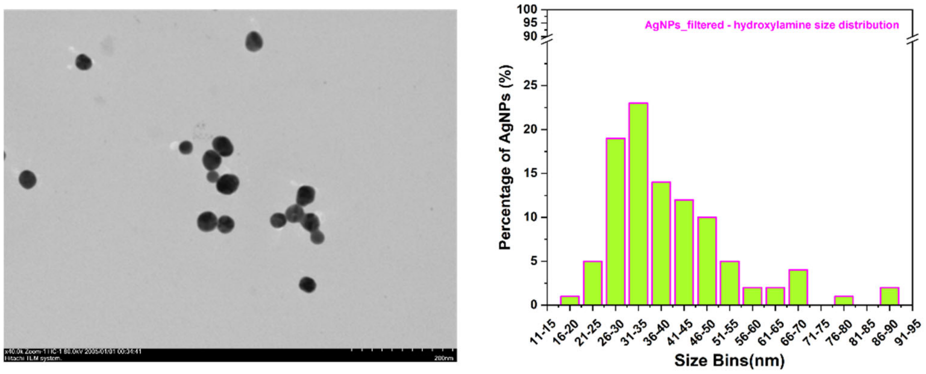


**Figure S3.** TEM image and size distribution graph of purified silver nanoparticles.

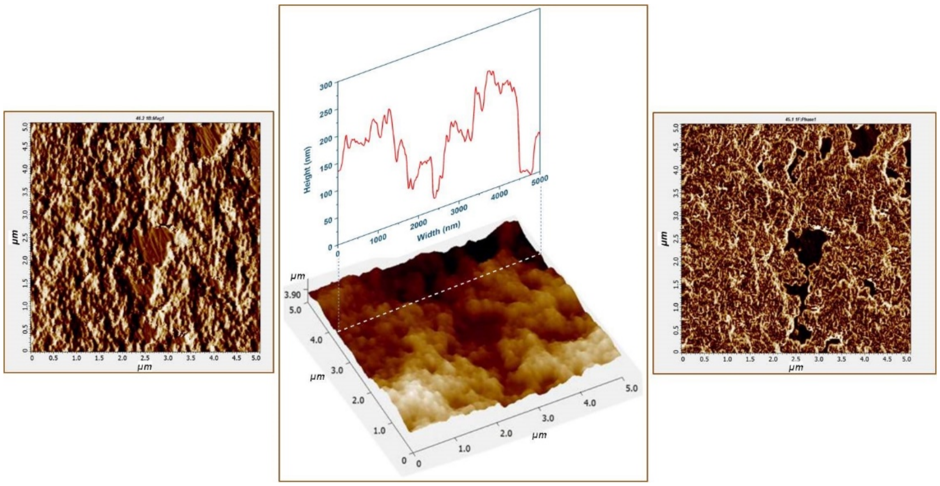


**Figure S4.** AFM topographic images of silver solid plasmonic substrates.

## Calculation of the EF

In our paper we have used rhodamine 6G (R6G) molecules for the calculation of substrates' enhancement factor (EF). According to a procedure proposed by Gupta and Weimar [1] the EF can be calculated by using the following equation:

$$EF = \frac{M_{Raman} \times S_{Surf} \times I_{Surf}}{M_{Surf} \times S_{Raman} \times I_{Raman}} \quad (1)$$

where $M_{Surf}$ and $M_{Raman}$ are the numbers of molecules dropped onto the solid plasmonic substrate (SERS measurements) and $CaF_2$ (Raman measurements), $S_{Surface}$ and $S_{Raman}$ are the geometrical areas of the molecular films and $I_{Surf}$ and $I_{Raman}$ are the SERS/Raman intensities of the most intense vibrational band that has been used for the calculation of EF (1508/1510 $cm^{-1}$). Both measurements were performed using a 50× objective and an excitation laser of 785 nm. In the case of Raman measurements, a 100% laser power was used, the acquisition time was 10 s and a number of 4 acquisition was recorded. For SERS measurements the laser power was set to 0.1%, all other conditions being identical. In order to improve the accuracy of EF calculation we measured the laser intensity on the sample surface in the two cases and the nominal values were 113 mW (100 % laser power) respectively 0.22 mW (0.1% laser power). The intensities of the 1508/1510 $cm^{-1}$ vibrational band, in both SERS and Raman spectra have been plotted in *kcounts*/(mW × s) units. For both measurements we have used $10^{-3}$ M aqueous solutions of R6G. The circular spots had a diameter of ~2 mm in both cases. The values of 1508/1510 $cm^{-1}$ intensities were 16.9 respectively 0.0054 kcounts/(mW × s).

Using these data, the EF of the here proposed solid substrates has a value of ~3 × $10^3$.

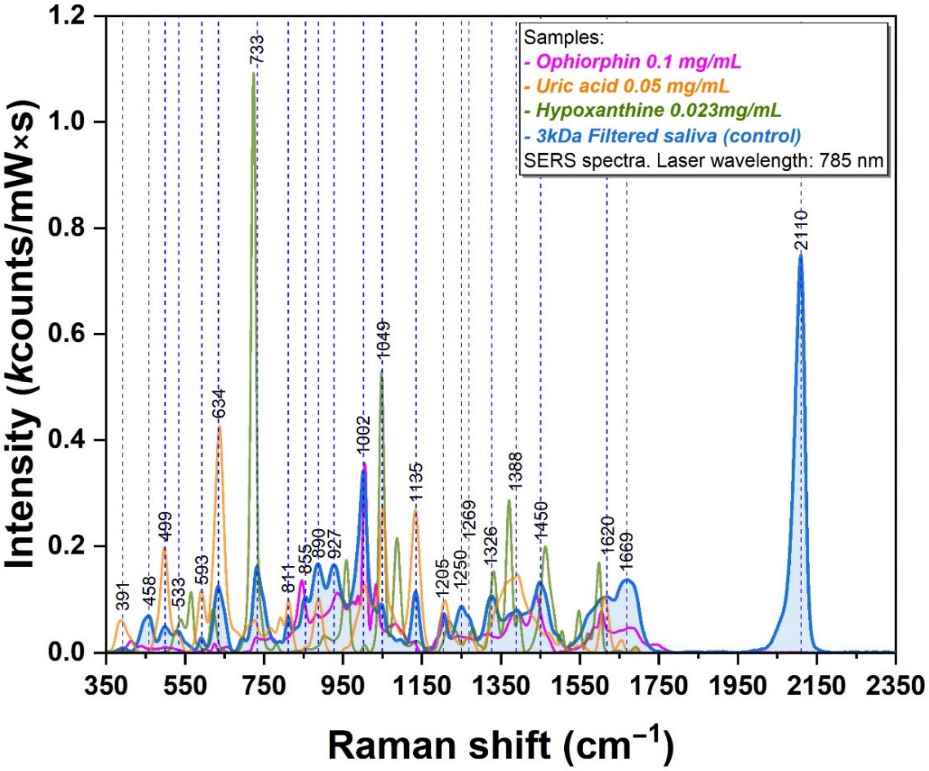


**Figure S5.** SERS spectrum of opiorphin, uric acid, hypoxanthine and salivary control probes using an excitation wavelength of 785 nm

**Supplementary Table S3.** Tentative assignment of Raman and SERS molecules

| Molecule | SERS vibrational band ($cm^{-1}$) | Contributions |
|---|---|---|
| **Amide I** | 1049, 1669 | [2–5] |
| **Amide III** | 1205 | [6,7] |
| **Amide VI** | 593 | [8] |
| **Ascorbic acid** | 593 | [8] |
| **Cholesterol ester** | 533 | [8] |
| **Collagen** | 811 | [9] |
| **D-galactosamine** | 890 | [9] |
| **D-mannose** | 1135 | [8,9] |
| **Glycine** | 890 | [10] |
| **Histidine** | 1135, 1269 | [10] |
| **Hypoxanthine** | 533, 634, 733, 1049, 1205 | [8,9,11,12] |
| **L-arginine** | 1326 | [10] |
| **L-glutamate** | 811, 1049 | [10] |
| **Lipids** | 1326, 1450 | [7,13] |
| **L-Phenylalanine** | 855, 1002 | [2–5,7–11,14–18] |
| **L-proline** | 855, 1049, 1269, 1326 | [10] |
| **L-serine** | 811 | [8] |
| **L-serine** | 1135, 1388 | [10] |
| **L-Tryptophan** | 458, 593, 890, 1049, 1205, 1326, 1388, 1620 | [3,8–10] |
| **L-tyrosine** | 634, 1049, 1135, 1205, 1269, 1326 | [4,7–11] |
| **L-valine** | 890, 1620 | [10] |

| | | |
|---|---|---|
| **Lysozyme** | 533 | [17] |
| **Nucleic acids** | 458, 890, 1135, 1269, 1326 | [6,10,19–21] |
| **Proteins** | 890, 927, 1049, 1205, 1450 | [3,4,6,7,13–15,17,22] |
| **Saccharides** | 458 | [17] |
| **Thiocyanate** | 2110 | [3,17,23] |
| **Uric acid** | 391, 499, 593, 634, 811, 890, 1002, 1049, 1135, 1269, 1388 | [11,12,22,24] |
| **Xanthine** | 1250 | [25] |

References


1. Gupta, R.; Weimer, W.A. High enhancement factor gold films for surface enhanced Raman spectroscopy. *Chem. Phys. Lett.* **2003**, *374*, 302–306, doi:10.1016/S0009-2614(03)00737-1.
2. Bankapur, A.; Zachariah, E.; Chidangil, S.; Valiathan, M.; Mathur, D. Raman Tweezers Spectroscopy of Live, Single Red and White Blood Cells. *PLoS One* **2010**, *5*, e10427, doi:10.1371/journal.pone.0010427.
3. Colceriu-Șimon; Hedeșiu; Toma; Armencea; Moldovan; Știufiuc; Culic; Țărmure; Dinu; Berindan-Neagoe; et al. The Effects of Low-Dose Irradiation on Human Saliva: A Surface-Enhanced Raman Spectroscopy Study. *Diagnostics* **2019**, *9*, 101, doi:10.3390/diagnostics9030101.
4. Cao, X.; Wang, Z.; Bi, L.; Zheng, J. Label-Free Detection of Human Serum Using Surface-Enhanced Raman Spectroscopy Based on Highly Branched Gold Nanoparticle Substrates for Discrimination of Non-Small Cell Lung Cancer. *J. Chem.* **2018**, *2018*, 1–13, doi:10.1155/2018/9012645.
5. Maiti, N.C.; Apetri, M.M.; Zagorski, M.G.; Carey, P.R.; Anderson, V.E. Raman Spectroscopic Characterization of Secondary Structure in Natively Unfolded Proteins: $\alpha$-Synuclein. *J. Am. Chem. Soc.* **2004**, *126*, 2399–2408, doi:10.1021/ja0356176.
6. Ryzhikova, E.; Ralbovsky, N.M.; Halámková, L.; Celmins, D.; Malone, P.; Molho, E.; Quinn, J.; Zimmerman, E.A.; Lednev, I.K. Multivariate Statistical Analysis of Surface Enhanced Raman Spectra of Human Serum for Alzheimer's Disease Diagnosis. *Appl. Sci.* **2019**, *9*, 3256, doi:10.3390/app9163256.
7. Buchan, E.; Kelleher, L.; Clancy, M.; Stanley Rickard, J.J.; Oppenheimer, P.G. Spectroscopic molecular-fingerprint profiling of saliva. *Anal. Chim. Acta* **2021**, *1185*, 339074, doi:10.1016/j.aca.2021.339074.
8. Wu, Q.; Qiu, S.; Yu, Y.; Chen, W.; Lin, H.; Lin, D.; Feng, S.; Chen, R. Assessment of the radiotherapy effect for nasopharyngeal cancer using plasma surface-enhanced Raman spectroscopy technology. *Biomed. Opt. Express* **2018**, *9*, 3413, doi:10.1364/BOE.9.003413.
9. Lin, D.; Pan, J.; Huang, H.; Chen, G.; Qiu, S.; Shi, H.; Chen, W.; Yu, Y.; Feng, S.; Chen, R. Label-free blood plasma test based on surface-enhanced Raman scattering for tumor stages detection in nasopharyngeal cancer. *Sci. Rep.* **2015**, *4*, 4751, doi:10.1038/srep04751.
10. De Gelder, J.; De Gussem, K.; Vandenabeele, P.; Moens, L. Reference database of Raman spectra of biological molecules. *J. Raman Spectrosc.* **2007**, *38*, 1133–1147, doi:10.1002/jrs.1734.
11. Știufiuc, G.F.; Toma, V.; Buse, M.; Mărginean, R.; Morar-Bolba, G.; Culic, B.; Tetean, R.; Leopold, N.; Pavel, I.; Lucaciu, C.M.C.M.; et al. Solid Plasmonic Substrates for Breast Cancer Detection by Means of SERS Analysis of Blood Plasma. *Nanomaterials* **2020**, *10*, 1212, doi:10.3390/nano10061212.
12. Tian, F.; Carvalho, L.F. das C. e S. de; Casey, A.; Nogueira, M.S.; Byrne, H.J. Surface-Enhanced Raman Analysis of Uric Acid and Hypoxanthine Analysis in Fractionated Bodily Fluids. *Nanomaterials* **2023**, *13*, 1216, doi:10.3390/nano13071216.
13. Tan, Y.; Yan, B.; Xue, L.; Li, Y.; Luo, X.; Ji, P. Surface-enhanced Raman spectroscopy of blood serum based on gold nanoparticles for the diagnosis of the oral squamous cell carcinoma. *Lipids Health Dis.* **2017**, *16*, 73, doi:10.1186/s12944-017-0465-y.
14. Lee, C.; Carney, R.P.; Hazari, S.; Smith, Z.J.; Knudson, A.; Robertson, C.S.; Lam, K.S.; Wachsmann-Hogiu, S. 3D plasmonic nanobowl platform for the study of exosomes in solution.

*Nanoscale* **2015**, *7*, 9290–9297, doi:10.1039/C5NR01333J.
15. Zhang, H.; Silva, A.C.; Zhang, W.; Rutigliano, H.; Zhou, A. Raman Spectroscopy characterization extracellular vesicles from bovine placenta and peripheral blood mononuclear cells. *PLoS One* **2020**, *15*, e0235214, doi:10.1371/JOURNAL.PONE.0235214.
16. Dingari, N.C.; Horowitz, G.L.; Kang, J.W.; Dasari, R.R.; Barman, I. Raman Spectroscopy Provides a Powerful Diagnostic Tool for Accurate Determination of Albumin Glycation. *PLoS One* **2012**, *7*, e32406, doi:10.1371/journal.pone.0032406.
17. Faur, C.I.; Dinu, C.; Toma, V.; Jurj, A.; Mărginean, R.; Onaciu, A.; Roman, R.C.; Culic, C.; Chirilă, M.; Rotar, H.; et al. A New Detection Method of Oral and Oropharyngeal Squamous Cell Carcinoma Based on Multivariate Analysis of Surface Enhanced Raman Spectra of Salivary Exosomes. *J. Pers. Med.* **2023**, *13*, 762, doi:10.3390/jpm13050762.
18. Tatischeff, I.; Larquet, E.; Falcón-Pérez, J.M.; Turpin, P.Y.; Kruglik, S.G. Fast characterisation of cell-derived extracellular vesicles by nanoparticles tracking analysis, cryo-electron microscopy, and Raman tweezers microspectroscopy. *J. Extracell. Vesicles* **2012**, *1*, doi:10.3402/JEV.V1I0.19179.
19. Stremersch, S.; Marro, M.; Pinchasik, B. El; Baatsen, P.; Hendrix, A.; De Smedt, S.C.; Loza-Alvarez, P.; Skirtach, A.G.; Raemdonck, K.; Braeckmans, K. Identification of individual exosome-like vesicles by surface enhanced raman spectroscopy. *Small* **2016**, *12*, 3292–3301, doi:10.1002/smll.201600393.
20. Otto, C.; van den Tweel, T.J.J.; de Mul, F.F.M.; Greve, J. Surface-enhanced Raman spectroscopy of DNA bases. *J. Raman Spectrosc.* **1986**, *17*, 289–298, doi:10.1002/jrs.1250170311.
21. Prescott, B.; Steinmetz, W.; Thomas, G.J. Characterization of DNA structures by laser Raman spectroscopy. *Biopolymers* **1984**, *23*, 235–256, doi:10.1002/bip.360230206.
22. Tefas, C.; Mărginean, R.; Toma, V.; Petrushev, B.; Fischer, P.; Tanțău, M.; Știufiuc, R. Surface-enhanced Raman scattering for the diagnosis of ulcerative colitis: will it change the rules of the game? *Anal. Bioanal. Chem.* **2021**, *413*, 827–838, doi:10.1007/s00216-020-03036-2.
23. Fălămaș, A.; Rotaru, H.; Hedeșiu, M. Surface-enhanced Raman spectroscopy (SERS) investigations of saliva for oral cancer diagnosis. *Lasers Med. Sci.* **2020**, 1–9, doi:10.1007/s10103-020-02988-2.
24. Iancu, S.D.; Cozan, R.G.; Stefancu, A.; David, M.; Moisoiu, T.; Moroz-Dubenco, C.; Bajcsi, A.; Chira, C.; Andreica, A.; Leopold, L.F.; et al. SERS liquid biopsy in breast cancer. What can we learn from SERS on serum and urine? *Spectrochim. Acta Part A Mol. Biomol. Spectrosc.* **2022**, *273*, 120992, doi:10.1016/j.saa.2022.120992.
25. Phyo, J. Bin; Woo, A.; Yu, H.J.; Lim, K.; Cho, B.H.; Jung, H.S.; Lee, M.-Y. Label-Free SERS Analysis of Urine Using a 3D-Stacked AgNW-Glass Fiber Filter Sensor for the Diagnosis of Pancreatic Cancer and Prostate Cancer. *Anal. Chem.* **2021**, *93*, 3778–3785, doi:10.1021/acs.analchem.0c04200.
